# Regulation of PI-3-Kinase and Akt Signaling in T Lymphocytes and Other Cells by TNFR Family Molecules

**DOI:** 10.3389/fimmu.2013.00139

**Published:** 2013-06-07

**Authors:** Takanori So, Michael Croft

**Affiliations:** ^1^Department of Microbiology and Immunology, Tohoku University Graduate School of Medicine, Sendai, Japan; ^2^Division of Immune Regulation, La Jolla Institute for Allergy and Immunology, La Jolla, CA, USA

**Keywords:** PI3K, AKT, TNFSF, TNFRSF, TRAF, signalosome

## Abstract

Activation of phosphoinositide 3-kinase (PI3K) and Akt (protein kinase B) is a common response triggered by a range of membrane-bound receptors on many cell types. In T lymphocytes, the PI3K-Akt pathway promotes clonal expansion, differentiation, and survival of effector cells and suppresses the generation of regulatory T cells. PI3K activation is tightly controlled by signals through the T cell receptor (TCR) and the co-stimulatory receptor CD28, however sustained and periodic signals from additional co-receptors are now being recognized as critical contributors to the activation of this pathway. Accumulating evidence suggests that many members of the Tumor Necrosis Factor receptor (TNFR) superfamily, TNFR2 (TNFRSF1B), OX40 (TNFRSF4), 4-1BB (TNFRSF9), HVEM (TNFRSF14), and DR3 (TNFRSF25), that are constitutive or inducible on T cells, can directly or indirectly promote activity in the PI3K-Akt pathway. We discuss recent data which suggests that ligation of one TNFR family molecule organizes a signalosome, via TNFR-associated factor (TRAF) adapter proteins in T cell membrane lipid microdomains, that results in the subsequent accumulation of highly concentrated depots of PI3K and Akt in close proximity to TCR signaling units. We propose this may be a generalizable mechanism applicable to other TNFR family molecules that will result in a quantitative contribution of these signalosomes to enhancing and sustaining PI3K and Akt activation triggered by the TCR. We also review data that other TNFR molecules, such as CD40 (TNFRSF5), RANK (TNFRSF11A), FN14 (TNFRSF12A), TACI (TNFRSF13B), BAFFR (TNFRSF13C), and NGFR (TNFRSF16), contribute to the activation of this pathway in diverse cell types through a similar ability to recruit PI3K or Akt into their signaling complexes.

## Introduction

The response of T lymphocytes to extrinsic stimuli has been known for many years to involve activation of phosphoinositide 3-kinase (PI3K) that results in a sustained rise in the lipid second messenger phosphatidylinositol (3,4,5)-trisphosphate (PIP_3_ or PI(3,4,5)P_3_), produced from phosphatidylinositol (4,5)-bisphosphate (PIP_2_ or PI(4,5)P_2_), and translocation of a subset of proteins containing pleckstrin homology (PH) domains to the plasma membrane, such as Akt (protein kinase B) and phosphoinositide-dependent kinase 1 (PDK1). Akt activity is regulated by the binding of PIP_3_ or phosphatidylinositol (3,4)-bisphosphate (PI(3,4)P_2_) to its PH domain, and by phosphorylation on threonine 308 by PDK1 and on serine 473 by the mammalian target of rapamycin complex 2 (mTORC2). Although PDK1 and mTORC2 may be central to Akt function, they likely have activities unrelated to Akt. For example, PDK1 also phosphorylates and activates other AGC protein kinases without binding to PIP_3_, such as 70 kDa ribosomal protein S6 kinases (S6Ks), 90 kDa ribosomal protein S6 kinases (RSKs), serum/glucocorticoid-regulated kinases (SGKs), and protein kinase Cs (PKCs) (Finlay and Cantrell, 2011). Importantly, the signaling network regulated by PI3K and Akt plays an integral role in promoting T cell activation, differentiation, and survival, and also participates in suppressing the induction of Foxp3-expressing regulatory T cells (T_reg_) that otherwise would limit the T cell response (Fruman and Bismuth, 2009; Huang and Sauer, 2010; Josefowicz et al., 2012; Okkenhaug, 2013). Activated Akt potentially regulates many downstream molecules, directly or indirectly through phosphorylation, that contribute to maximizing the T cell response. These include blocking the activity of forkhead box O (Foxo) transcription factors such as Foxo1 that promote differentiation of inducible T_reg_, suppressing the activity or expression of pro-apoptotic molecules such as Bad and Bim, antagonizing the expression of cell cycle inhibitor proteins, and promoting T cell functionality and survival by increasing glucose uptake and glycolysis, and through augmenting IκB Kinase and NF-κB activity.

**Figure 1:**
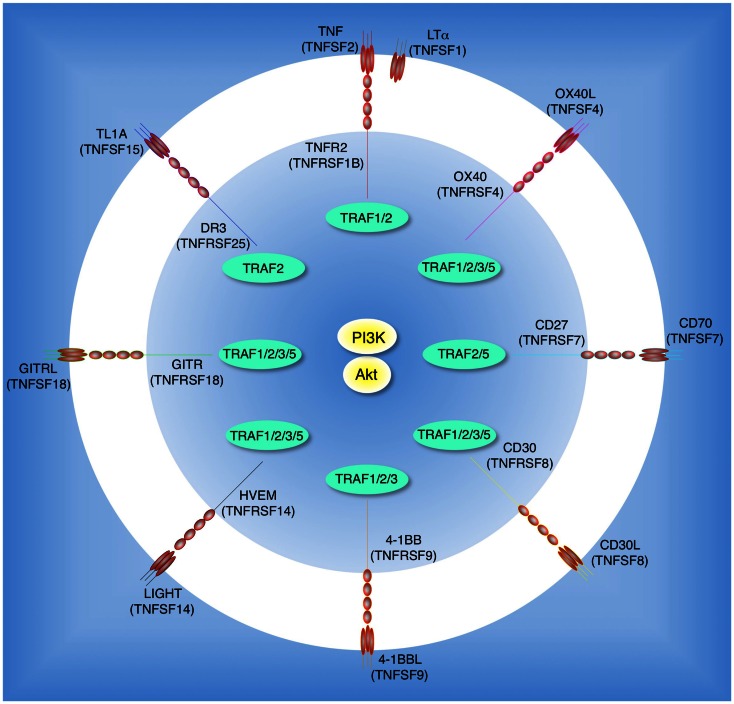
**Tumor necrosis factor receptor family molecules that possibly activate the PI3K-Akt pathway in T cells**. Molecular interactions between TNF receptor superfamily (TNFRSF) members and TNF ligand superfamily (TNFSF) members: TNFR2 (TNFRSF1B) and TNF (TNFSF2) or LTα (TNFSF1); OX40 (TNFRSF4) and OX40L (TNFSF4); CD27 (TNFRSF7) and CD70 (TNFSF7); CD30 (TNFRSF8) and CD30L (TNFSF8); 4-1BB (TNFRSF9) and 4-1BBL (TNFSF9); HVEM (TNFRSF14) and LIGHT (TNFSF14); GITR (TNFRSF18) and GITRL (TNFSF18); DR3 (TNFRSF25) and TL1A (TNFSF15). Interactions between TNFR-associated factors (TRAFs) and TNFRSF molecules are indicated in the inner circle. CD27, CD30, and GITR have yet to be described to promote activation of PI3K or Akt, but this is likely given their overlapping TRAF-binding capacity.

The range of membrane receptors that participate in triggering this PI3K/Akt axis in T cells may have been underappreciated. Recognition of antigen by the T cell receptor (TCR) in the context of signaling from the co-stimulatory receptor CD28 has long been known to promote PI3K activity. CD28 is constitutively expressed on T cells, and engagement by B7 molecules (CD80 and CD86) directly recruits the p85 regulatory subunit of PI3K (p85 PI3K) through a pYMNM (phospho-Tyr-Met-Asn-Met) motif located in CD28’s cytoplasmic tail (Pages et al., 1994). The overall signaling activity of CD28, including through the PI3K and Akt pathway, participates in the initial activation and division of T cells in many situations, although extensive studies have also suggested that the interaction with p85 is dispensable for many functions of CD28 in naïve T cells (Fruman and Bismuth, 2009). As well as CD28 and related molecules like ICOS, many additional receptors on T cells may contribute to PI3K and Akt activity. Sustained and periodic signaling from these receptors over time is increasingly being recognized as vital for continued T cell differentiation and survival, further suppression of T_reg_ development, the generation of memory, and the reactivation of memory T cells. Members of the tumor necrosis factor receptor (TNFR) superfamily constitute many of these receptors (Croft, 2003, 2009; Watts, 2005; So et al., 2006). Although all of the TNFR family members discussed in this review are strong activators of NF-κB, and NF-κB certainly plays a role in many of the functional consequences of triggering these receptors, increasing evidence suggests that their ability to also target PI3K and Akt may be integral to their function. In T cells, these receptors include TNFR2 (TNFRSF1B), OX40 (TNFRSF4), 4-1BB (TNFRSF9), HVEM (TNFRSF14), and DR3 (TNFRSF25), which are either constitutively expressed or induced after activation (Figure 1). There are several other TNFR molecules that control T cell function, such as CD27 (TNFRSF7), CD30 (TNFRSF8), and GITR (TNFRSF18), that have yet to be described to promote activation of PI3K or Akt, however it is highly likely that they also have the ability to target this pathway (Figure 1). This review summarizes recent findings on the potential importance of TNFR family signaling in stimulating the PI3K-Akt pathway in T cells as well as in other cell types, and discusses the likely mechanism of how TNFR family molecules organize signalosomes on the membrane to sustain lipid signaling. The initial sections will present a brief overview of reported activities of TNFR family members on T cells that have been described to augment PI3K or Akt activity. We will then discuss the potential molecular connections that allow these molecules to link to PI3K or Akt, and lastly review PI3K and Akt related activation by other TNFR family members in non-T cells.

## Regulation of T Cell Co-Signaling by TNFRSF Members: TNFR2, OX40, 4-1BB, HVEM, and DR3

### TNFR2 (TNFRSF1B)

TNFR2 is mainly expressed in cells of the immune system including T cells. The ligand TNF (TNFSF2) is produced by activated macrophages, T cells, and many other cell types, and exists as a transmembrane trimer whose proteolysis also leads to a soluble form. TNFR2 is more efficiently triggered by transmembrane TNF than by soluble TNF (Grell et al., 1995; Faustman and Davis, 2010). TNFR2 is constitutively expressed on T cells and increases its expression after T cell activation. Interaction of TNF with TNFR2 is co-stimulatory to TCR-mediated T cell activation and effector T cell differentiation (Kim and Teh, 2001, 2004; Aspalter et al., 2003; Kim et al., 2006) and TNFR2-deficient T cells possess a defect in survival during the early phase of clonal expansion that correlates with a defect in survivin, Bcl-2, and Bcl-_XL_ expression (Kim and Teh, 2004; Kim et al., 2006). Importantly, TNFR2 was found to sustain Akt activity in T cells stimulated through the TCR and CD28. Given the described activities of PI3K-Akt in promoting expression of the aforementioned anti-apoptotic and cell cycle related molecules in various cell types including T cells, this data suggested that TNFR2 triggered Akt signaling may have participated in regulating expansion and survival of these effector-type T cells (Kim and Teh, 2004). The differentiation of effector T cells from a naïve population is counter to the differentiation of regulatory T cells (iT_reg_), and activation of PI3K and Akt has been shown to block induction of Foxp3 and iT_reg_ development (Haxhinasto et al., 2008; Sauer et al., 2008). In line with this, neutralization of TNF has also recently been found to enhance development of iT_reg_ cells (Zhang et al., 2013). TGF-β-induced Smad3 phosphorylation directs transcription of Foxp3 and formation of iT_reg_, and phosphorylation of Akt through TNF-TNFR2 interaction was described to facilitate Akt-Smad3 interaction and suppress Foxp3 expression, potentially explaining in part why TNF would block iT_reg_ differentiation (Zhang et al., 2013).

Substantiating that Akt is a general target of TNF signaling, Akt phosphorylation has also been shown to be enhanced through TNFR1 and/or TNFR2 in various cell types, such as HEK293 cells (Ozes et al., 1999), HeLa cells (Ozes et al., 1999; Pastorino et al., 1999), HepG2 cells (Reddy et al., 2000), U937 cells (Reddy et al., 2000), endothelial cells (Zhang et al., 2003a), fibroblasts (Hanna et al., 1999; Zhang et al., 2001), myocytes (Hiraoka et al., 2001), cortical neurons (Marchetti et al., 2004), and hepatocytes (Osawa et al., 2001).

### OX40 (TNFRSF4)

OX40 is induced on activated T cells while its ligand, OX40L (TNFSF4), is inducible on professional antigen-presenting cells (APCs). OX40-OX40L interactions positively regulate conventional T cell responses and can negatively affect T_reg_ differentiation (So et al., 2008; Croft et al., 2009; Croft, 2010; Ishii et al., 2010). OX40 functions later than CD28, and potentially later than TNFR2, providing signals to promote continued division and survival, and hence clonal expansion of effector and memory T cells (Gramaglia et al., 2000). OX40 signaling was shown to augment and sustain PI3K-Akt activity when antigen was presented to T cells, again correlating with its ability to promote continued expression of molecules that control cell cycle progression as well as anti-apoptotic Bcl-2 family members (Rogers et al., 2001; Song et al., 2004, 2005). Importantly, a dominant-negative version of Akt reproduced many of the defects associated with a lack of OX40 expression, and introduction of a constitutively active version of Akt into T cells that lacked OX40 almost fully reversed the defect in clonal expansion and survival exhibited by these T cells (Rogers et al., 2001; Song et al., 2004, 2005). OX40 signaling also antagonizes the differentiation of Foxp3+ or IL-10+ iT_reg_ (Ito et al., 2006; So and Croft, 2007; Vu et al., 2007). No formal proof has been provided that OX40 inhibition of Foxp3 and iT_reg_ development is mediated in part by Akt activation, but again this is likely.

### 4-1BB (TNFRSF9)

4-1BB is another inducible molecule on activated T cells that can be triggered by 4-1BBL (TNFSF9) expressed on activated APCs (So et al., 2008; Snell et al., 2011; Vinay and Kwon, 2012). 4-1BB ligation can again promote T cell clonal expansion, differentiation, and expression of cytokines, and can enhance the survival of effector and memory T cells through upregulation of Bcl-_XL_. 4-1BB signaling also has the ability to inhibit TGF-β-driven conversion of naïve CD4^+^ T cells into iT_reg_ either through direct activity or indirectly via upregulation of IFNγ production (Madireddi et al., 2012). 4-1BB was found to promote phosphorylation of Akt in T cells, and proliferative responses mediated by 4-1BB were blocked by a PI3K inhibitor, coincident with suppressing cyclin expression and promoting the cell cycle regulatory molecules p27^kip1^ (Lee et al., 2002, 2003). Some studies suggested that 4-1BB induced the anti-apoptotic Bcl-2 family molecules, Bcl-2 and Bcl-xL, in murine T cells in a PI3K-Akt independent manner (Lee et al., 2002, 2003). However, suppression of apoptosis and induction of c-FLIP_short_ and Bcl-_XL_ by 4-1BB in human peripheral blood T cells was blocked by targeting PI3K or Akt (Starck et al., 2005).

### HVEM (TNFRSF14)

HVEM can interact with a number of different ligands, however its primary activating ligand in the TNF family is LIGHT (TNFSF14). HVEM is widely expressed on many cell types, including being constitutive on T cells. LIGHT in contrast is inducible on T cells as well as certain APCs such as DC and B cells upon activation (Steinberg et al., 2011; Ware and Sedy, 2011). Ligation of HVEM by LIGHT provides stimulatory signals that additionally can impact activation, differentiation, or survival of T cells. In line with HVEM controlling Akt activation at later stages of T cell responses, HVEM-deficient T cells were shown to display reduced Akt activity at the peak of the effector response that correlated with defective expression of Bcl-2 and reduced T cell survival. Furthermore, the defect in T cell survival was rescued by ectopic expression of an active form of Akt (Soroosh et al., 2011).

Substantiating Akt as a downstream target of HVEM, the ability of LIGHT to induce macrophage migration and vascular smooth muscle cell proliferation also correlated with activation of PI3K and Akt (Wei et al., 2006), and LIGHT was found to promote PI3K-Akt phosphorylation in osteoclast precursor cells, supporting osteoclast differentiation (Hemingway et al., 2013).

### DR3 (TNFRSF25)

DR3 is constitutively expressed by T cells and is upregulated following T cell activation, while TL1A (TNFSF15), the ligand for DR3, is induced in APCs (Meylan et al., 2011). Interaction of TL1A with DR3 also provides costimulatory signals to T cells in concert with antigen/TCR signaling and this can contribute to enhanced production of pro-inflammatory cytokines, and increased clonal expansion and differentiation of T cells. Although no studies have been conducted as yet on conventional T cells, ligation of DR3 was shown to promote T_reg_ proliferation that was blocked by an Akt inhibitor (Schreiber et al., 2010). Stimulation of DR3-expressing human acute monocytic leukemia THP-1 cells with TL1A, or anti-DR3 antibodies, also induced phosphorylation of Akt concomitant with upregulation of expression of βig-h3, an extracellular matrix protein. This was blocked by an inhibitor of PI3K and inhibitors of PKC, suggesting that PKC activation by DR3 may be involved in PI3K-Akt activation via this receptor in this cell type (Lee et al., 2010). Lastly, E-selectin (CD62E) has been suggested to be an alternate ligand for DR3, and E-selectin was found to activate the PI3K-Akt pathway via DR3 in HT29 colon carcinoma cells (Porquet et al., 2011).

## TNF Receptor Oligomerization, Membrane Lipid Microdomains, and the T Cell TNFR Signalosome

Although the studies described above show that TNFR2, OX40, 4-1BB, HVEM, and DR3 can enhance PI3K and Akt activation in T cells or other cell types, a primary question is whether this is a direct activity, or indirect through modulating or enhancing signaling through other non-TNFR molecules including the TCR or CD28. Moreover, as these TNFR molecules do not have obvious PI3K-binding motifs, similar to the pYMNM motif of CD28, it is not clear how they would directly link to PI3K or Akt or how the connection with the lipid mediators is facilitated.

TNF family ligands share the TNF homology domain (THD), which binds to cysteine-rich domains (CRDs) of the TNF family receptors. TNF ligands are synthesized as either membrane-bound or soluble trimeric proteins. Many biochemical and functional studies show that the transmembrane ligands can robustly activate receptors whereas the soluble trimeric ligands differ in their ability to be activating molecules. Some TNFR molecules, such as TNFR1 and CD40, are thought to be pre-clustered at the cell surface in the absence of their cognate TNF ligand (Chan, 2007), which likely aids their ability to respond to the soluble ligand. This is exemplified by TNF, which is highly active in soluble form when recognizing TNFR1. In contrast, studies of trimers of molecules such as OX40L and 4-1BBL have suggested they do not have functional effects when soluble, implying their receptors are not pre-assembled into clusters. However, artificially generated oligomerized versions of soluble trimeric ligands, including OX40L, 4-1BBL, and GITRL work as highly efficient agonists in T cells and other cell types (Haswell et al., 2001; Zhang, 2004; Stone et al., 2006; Muller et al., 2008; Zhou et al., 2008; Wyzgol et al., 2009). Other variants on this theme are molecules like APRIL whose soluble form can be oligomerized naturally through interaction with polysaccharide side chains of heparin sulfate proteoglycans, allowing effective signals through its receptors TACI or BCMA (Ingold et al., 2005; Kimberley et al., 2009); or BAFF that also binds to TACI, and is unable to activate this receptor as a single trimer, but can assemble as an ordered structure comprising 20 trimers (60-mer) and then gains the ability to be a strong TACI agonist (Liu et al., 2002; Bossen et al., 2008). In sum, these results suggest that oligomerization of most TNFR molecules, beyond the basic trimer complex that would be formed after ligation of a single trimeric ligand, is a prerequisite for efficient recruitment and activation of signaling moieties.

All TNFR family molecules also promote intracellular kinase activation at least in part through adaptor proteins called TNFR-associated factors (TRAFs). For example, TNFR2 has the potential ability to recruit and/or directly bind TRAFs 1 and 2 (Rothe et al., 1994); OX40: TRAFs 1, 2, 3, and 5 (Kawamata et al., 1998); 4-1BB: TRAFs 1, 2, and 3 (Jang et al., 1998); HVEM: TRAFs 1, 2, 3, and 5 (Marsters et al., 1997); and DR3: TRAF2 (Chinnaiyan et al., 1996). TRAF proteins already can exist as trimers in the cytosol before binding to the cytoplasmic tails of TNF receptors (Park et al., 1999), suggesting that oligomerization of the receptors will then additionally result in oligomerized scaffolds of at least one, but more likely multiple, TRAF molecules.

Another facet that might be important to the ability of TNFR family molecules to link to PI3K and Akt directly is the regulated movement of TNFR oligomers into detergent-insoluble cholesterol- and sphingolipid-rich plasma membrane microdomains (DIM or lipid rafts). Here the spatiotemporal regulation of protein–protein interactions and dynamic protein networks may orchestrate to allow any biological outcome (Dykstra et al., 2003; Viola and Gupta, 2007). DIM are estimated as <20 nm diameter in a living cell (Eggeling et al., 2009), indicating that molecules that translocate into DIM are likely condensed into a small area. Although not investigated for many TNFR molecules to date, particularly in T cells, several members of the family have been visualized to concentrate in DIM after stimulation by their ligands including OX40 (So et al., 2011a,b) and 4-1BB (Nam et al., 2005). Therefore, translocation into lipid-rich microdomains might be a common and important feature of the TNFR family. Moreover, TRAF2 can interact with Filamin-A, which functions as a scaffold for DIM formation (Leonardi et al., 2000; Arron et al., 2002), and with Caveolin-1, which is a component of DIM (Feng et al., 2001), suggesting that recruitment of this molecule might promote or maintain localization of TNFR molecules in these lipid-rich areas. TRAF2 binding is shared by all TNFR family molecules that have been described to co-stimulate T cells (Figure 1), including those shown to date to promote PI3K and Akt activation (see above), implying TRAF2 may be a critical link to PI3K and/or Akt. Perhaps of equal significance, PIP_2_ is enriched and constitutively associated with DIM, and at least in T cells, a proportion of total cellular PI3K and PDK1 are constitutively associated with detergent-insoluble fractions (Pike and Casey, 1996; Dykstra et al., 2003; So et al., 2011a). Therefore, it is reasonable to suggest that these lipid-rich microdomains are likely to play a critical role in triggering PI3K-Akt signaling by facilitating the localization of oligomerized TNFR and TRAF molecules with PI3K, PIP_2_, and PDK1 (Lasserre et al., 2008). This would enhance the likelihood of PIP_3_ production, and membrane recruitment and phosphorylation of Akt, assuming PI3K can be activated.

**Figure 2:**
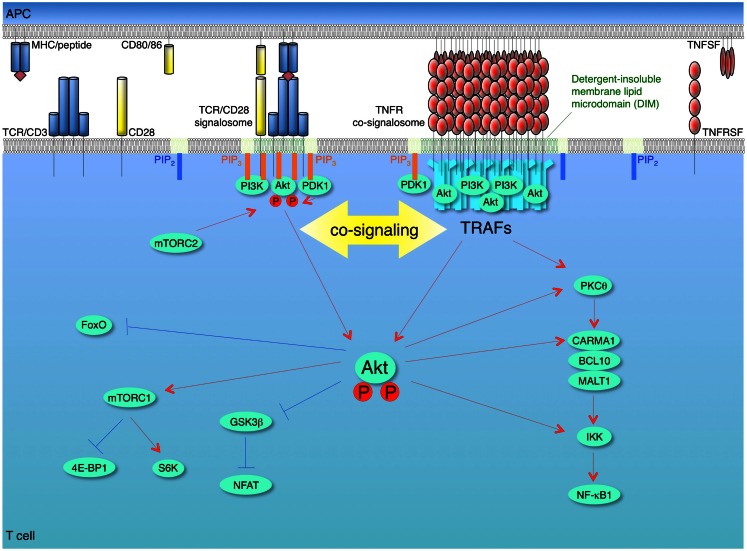
**Model of synergy between TCR/CD28 and TNFR signalosomes for activation of the PI3K-Akt pathway in T cells**. T cells are activated firstly by recognition of antigen by the T cell receptor (TCR)/CD3 complex when it is displayed by the major histocompatibility complex (MHC) on antigen-presenting cells (APCs). The second co-stimulatory signal is delivered through CD28 by interaction with its ligands CD80 and/or CD86. These combined signals can activate phosphoinositide 3-kinase (PI3K), which leads to conversion of PIP_2_ into PIP_3_ at the plasma membrane. The pleckstrin homology (PH) domain containing proteins, Akt (protein kinase B) and phosphoinositide-dependent kinase 1 (PDK1), are recruited to the membrane PIP_3_, and then Akt is phosphorylated by PDK1 and by the mammalian target of rapamycin complex 2 (mTORC2). This promotes translocation of Akt from membrane to cytosol, thereby allowing regulation of downstream pathways through phosphorylation of target molecules, such as glycogen synthase kinase 3β (GSK3β), forkhead box O (Foxo), and IκB kinase (IKK). Akt phosphorylates and inactivates two negative regulators of mTORC1, tuberous sclerosis complex 2 (TSC2) and proline-rich Akt substrate of 40 kDa (PRAS40), which results in activation of mTORC1. Akt contributes to NF-κB activation through phosphorylation of IKK and interaction with protein kinase C θ (PKCθ) or caspase-recruitment domain (CARD)-membrane-associated guanylate kinase (MAGUK) protein 1 (CARMA1). After recognition of trimeric TNF ligand superfamily (TNFSF) molecules on APCs, TNF receptor superfamily (TNFRSF) molecules on T cells are trimerized and oligomerized and recruit trimeric TNFR-associated factors (TRAFs) to their cytoplasmic TRAF-binding motifs. The TNFSF-TNFRSF complex then translocates into detergent-insoluble membrane lipid microdomains (DIM). The TNFRSF-TRAF superclusters recruit and allow the efficient accumulation of PI3K and Akt in concentrated depots in close proximity to the TCR/CD28 signalosome, which results in a quantitative contribution of TNFR signalosomes to enhancing and sustaining PI3K and Akt activation triggered by the TCR/CD28 signalosome. The TNFR signalosomes also can promote activation of NF-κB irrespective of TCR/CD28 signaling. Red lines show activating signals, blue lines show inhibitory signals.

Only studies of one molecule to date have shown a direct link of a TNFR family molecule to PI3K and Akt in T cells. However, there is strong rationale that the findings will be generalizable. We clearly demonstrated in several studies that OX40 signaling strongly synergizes with antigen signals to augment Akt activity in recently activated or effector T cells (Song et al., 2004; So et al., 2011a,b). After interaction of OX40 with transmembrane OX40L, OX40 moved into DIM and immunoprecipitation experiments revealed that it organized a signalosome containing many molecules including TRAF2, the IKK complex, and PKCθ and the CARMA1-BCL10-MALT1 complex, that regulate NF-κB1 activation, and also including p85 PI3K and Akt (So et al., 2011a,b). The formation of this complete OX40 signalosome in T cells was dependent on TRAF2 and on translocation of OX40 into DIM, but independent of antigen/TCR stimulation (So et al., 2011a,b). Moreover, in the absence of TRAF2 or by disrupting DIM, OX40 could not complex with either PI3K or Akt. Interestingly, OX40 was unable to induce significant cellular phosphorylation of PI3K, PIP_3_ accumulation, or Akt activation, unless antigen was presented to the T cells, even though antigen/TCR signaling had no obvious impact on recruitment of PI3K or Akt to the OX40 signalosome. The explanation for this was not clear, but we only found a moderate amount of PDK1 associated with the OX40 complex suggesting that this might in part contribute to the inability of OX40 ligation in isolation to lead to phosphorylation of Akt, and why antigen recognition was essential. However, more recent data have shown that OX40 associates with an E3 ligase that appears to limit its ability to activate Akt in T cells (Croft, unpublished). This then suggests that OX40 does possess the capacity to activate Akt independently of other receptors, but regulatory elements may keep this ability in check in T cells providing control over this aspect of OX40 biology to the TCR. Importantly, these data then imply that OX40 functions in T cells by quantitatively enhancing the amount of PI3K and Akt that is available to be activated in the lipid-rich microdomain environment. We hypothesize that the higher ordered oligomerized TNFR-ligand modules that are organized in DIM of T cells essentially offer functional hot spots of concentrated PI3K and Akt in the vicinity of the TCR/CD28 signalosome (Figure 2). These data also highlight that TRAF adaptors are likely to play critical roles in linking the TNFR family to PI3K and Akt in T cells. Our studies show that TRAF2 is important for OX40 to recruit both PI3K and Akt into its signaling complex, but whether TRAF2 directly binds one or both molecules is not yet clear. Other TRAFs, particularly TRAF6, may be also critical as described below in non-T cells, although how much this might vary from a T cell to another cell type is also not clear.

## Regulation of PI3K and AKT by TNFRSF Members in Non-T Cells: CD40, RANK, FN14, TACI/BAFFR, and NGFR

### CD40 (TNFRSF5)

Signaling through CD40 after ligation by CD40L (TNFSF5) is important for promoting the activation, division, and maturation of APCs, and isotype switching of B cells (Graham et al., 2010; Gommerman and Summers deLuca, 2011). CD40 can directly bind to TRAFs 1, 2, 3, 5, and 6 (Pullen et al., 1998) and CD40 engagement leads minimally to translocation of CD40, TRAF2, TRAF3, and TRAF6 into DIM where CD40 activates downstream signaling cascades (Hostager et al., 2000; Vidalain et al., 2000; Arron et al., 2001). Cross-linking CD40 has been found to activate PI3K in the Daudi human B lymphoblastoid line (Ren et al., 1994), and promote Akt phosphorylation, downregulation of p27^kip1^, and upregulation of Bcl-_XL_, in primary murine B cells that was blocked by a PI3K inhibitor (Andjelic et al., 2000). CD40-induced proliferation and survival of B cells deficient in a negative regulatory adaptor molecule was also suppressed by introduction of dominant-negative Akt (Aiba et al., 2006). After triggering CD40 on murine bone marrow-derived DCs, p85 PI3K was furthermore found to be recruited to CD40 correlating with enhanced Akt activation (Arron et al., 2001). Similarly, CD40L induced Akt phosphorylation and survival in human monocyte-derived DCs that was blocked with a PI3K inhibitor (Yu et al., 2004). In other cells, stimulation of CD40 on human microvascular endothelial cells also induced PI3K and Akt phosphorylation, concomitant with an increase in cell survival and proliferation, and these functional activities were suppressed by PI3K inhibitors and a dominant-negative version of Akt (Deregibus et al., 2003).

Although the adaptors required for CD40 to connect to PI3K and Akt have not been investigated in every situation, several pieces of evidence suggest TRAF6 and/or TRAF2 are crucial. For example, CD40 was shown to block apoptosis induced by the Fas death receptor in a PI3K and Akt dependent manner, and this was abrogated in B cells that were deficient in TRAF6 (Benson et al., 2006). A CD40 signalosome containing TRAF2, TRAF6, and p85 PI3K was also visualized in endothelial cells (Deregibus et al., 2003) and a signalosome of TRAF6 and PI3K in DCs (Arron et al., 2001). Lastly, fibroblasts lacking TRAF2 or TRAF6 displayed impaired Akt phosphorylation that was triggered by CD40 engagement (Davies et al., 2005).

### RANK (TNFRSF11A)

RANK interactions with RANKL (TNFSF11) regulate bone remodeling, lymph node formation, establishment of the thymic microenvironment, and mammary gland development during pregnancy (Leibbrandt and Penninger, 2008). RANK has the potential to recruit TRAFs 1, 2, 3, 5, and 6 (Wong et al., 1998; Darnay et al., 1999) and triggering of RANK with RANKL has been shown to promote activation of the PI3K-Akt pathway in osteoclasts and DCs. Similar to CD40, RANK was visualized to induce a signalosome containing TRAF6 and p85 PI3K in these cell types. PP1, an inhibitor for Src family kinases, inhibited Akt phosphorylation mediated by RANK, indicting that c-Src is an upstream regulator of PI3K. In accordance, the kinase activity of c-Src was upregulated in the RANK signalosome and RANK was found unable to activate Akt without the enzymatic activity of c-Src (Wong et al., 1999; Arron et al., 2001; Xing et al., 2001).

TRAF6 again may be critical for the ability of RANK to target the PI3K-Akt pathway, and certain elements might also limit Akt activation. Upon stimulation of RANK in the RAW264.7 monocyte/macrophage cell line, phosphorylation of PI3K and Akt was upregulated and this was further amplified by introduction of dominant-negative SHP-1. TRAF6 was found to interact with RANK and SHP-1, and SHP-1 antagonized the association between RANK and TRAF6 (Zhang et al., 2003b). Similar to other TNFR molecules, RANK may also function in the context of DIM. After engagement by RANKL in osteoclasts, TRAF6 was shown to translocate into DIM where c-Src is constitutively resident, and disruption of DIM reduced Akt activation and concomitantly blocked osteoclast differentiation, survival, and bone resorption activity (Ha et al., 2003).

### FN14 (TNFRSF12A)

FN14 is another TNFR molecule. It is expressed on epithelial cells, endothelial cells, and other non-hematopoietic cells and engages TWEAK (TNFSF12) and promotes a number of differentiation activities depending on the cell type. Stimulation of FN14 on mouse osteoblastic MC3T3-E1 cells induced Akt phosphorylation and RANTES production in a PI3K-dependent manner (Ando et al., 2006). TWEAK also promoted expression of ICAM-1 and VCAM-1 on human gingival fibroblasts that was blocked with an inhibitor of PI3K (Hosokawa et al., 2006), and Akt phosphorylation and matrix metalloprotease-9 (MMP-9) expression in mouse C2C12 myotubes was suppressed by targeting PI3K or introducing a dominant-negative version of Akt (Kumar et al., 2009). Similar results were also reported with TWEAK activation of FN14 on: renal tubular epithelium cells where PI3K inhibitors prevented upregulation of cyclin D1 and cell proliferation (Sanz et al., 2009); cardiomyocytes where FN14-mediated proliferation was also blocked by interfering with PI3K activity (Novoyatleva et al., 2010); and human gingival fibroblasts where the PI3K-Akt pathway contributed to induction of CCL20 (Hosokawa et al., 2012). FN14 has the potential to directly recruit and bind TRAFs 1, 2, 3, and 5 (Brown et al., 2003) but no studies to date have attempted to link a specific TRAF to the ability of FN14 to phosphorylate and activate PI3K or Akt.

### TACI (TNFRSF13B)/BAFFR (TNFRSF13C)/BCMA (TNFRSF17)

TACI, BAFFR, and BCMA are mainly expressed on B cells and play critical roles in survival of B cells at distinct stages of development by engaging APRIL (TNFSF13) and/or BAFF (TNFSF13B) (Rickert et al., 2011). BAFFR can activate the PI3K-Akt pathway in mature B cells (Patke et al., 2006; Otipoby et al., 2008; Woodland et al., 2008) and BAFF-mediated proliferative and survival responses were defective in B cells lacking p110δ PI3K (Henley et al., 2008). PKCβ was found to interact and directly phosphorylate Akt on serine 473 after ligation of BAFFR, with Akt phosphorylation being greatly reduced in PKCβ-deficient B cells (Patke et al., 2006). Follicular lymphoma B cells were additionally found to respond to APRIL-TACI stimulation by phosphorylating p85 PI3K, Akt, mTOR, 4E-BP1, and p70S6K, and PI3K inhibitors blocked these APRIL-induced activities and cellular proliferation (Gupta et al., 2009).

Stimulation of human myeloma cells expressing TACI, BAFFR, and BCMA with BAFF or APRIL has also been shown to activate the PI3K-Akt pathway concomitant with protection against apoptosis (Moreaux et al., 2004); and lastly human adipose-derived stem cells additionally phosphorylated Akt after exposure to APRIL or BAFF (Zonca et al., 2012). Similar to other TNFR family members, TACI, BAFFR, and BCMA may directly recruit and bind TRAFs 2, 5, and 6 (Xia et al., 2000), TRAFs 2 and 3 (Xu and Shu, 2002), and TRAFs 1, 2, 3, 5, and 6 (Hatzoglou et al., 2000; Shu and Johnson, 2000), respectively, suggesting that TRAF2 and/or 6 may again mediate PI3K-Akt signaling, although no studies have addressed this as yet.

### NGFR (TNFRSF16, p75^NTR^)

Lastly, NGFR that is mainly expressed on neurons and glia during development of the central nervous system, and is induced after many types of nervous system injury, also appears to utilize PI3K and Akt for certain activities. NGFR only binds neurotrophins [nerve growth factor (NGF); brain-derived neurotrophic factor (BNDF); and neurotrophin-3 and -4 (NT-3 and -4)]. NGFR may primarily work as a co-receptor and cooperates with the Trk receptor tyrosine kinase family (Trk-A, -B, and -C), Sortilin-family receptors, and Nogo receptor/Lingo-1 (Ibanez and Simi, 2012). Many studies (e.g., refs Soltoff et al., 1992; Yao and Cooper, 1995; Jackson et al., 1996; Vaillant et al., 1999; Takano et al., 2000) have shown that NGF activates PI3K and induces PIP_3_ production, and that the Akt pathway works as a key regulator of neurotrophin-induced neuronal survival. NGFR signaling is initiated in caveolae, which are a special type of DIM and serve as signaling platforms (Bilderback et al., 1999; Huang et al., 1999), but the contribution of individual TRAF molecules to activation of PI3K and Akt has not yet been reported. NGFR can directly bind TRAFs 2 and 6 (Khursigara et al., 1999; Ye et al., 1999), and TRAF6 recruitment has been suggested to be essential for signal transduction activity (Vilar et al., 2009), again implying this receptor may connect to the PI3K-Akt pathway via similar TRAF adaptors as other members of the TNFR family.

## Conclusion

In conclusion, many stimulatory TNFR family members have been reported to augment PI3K and Akt activation in diverse cell types, suggesting that this pathway can be a major contributor to the functional effects mediated by these molecules. After interaction with their transmembrane, and in some cases soluble, TNF family ligands, TNFR molecules oligomerize and organize signalosomes in membrane lipid microdomains. The cytoplasmic domain of TNFR family members does not have the consensus motif that can directly bind PI3K. Rather, TNFR molecules bind to overlapping but distinct subsets of TRAF adaptor proteins, and these adaptors initiate many of the signals delivered by the receptors. Increasing evidence suggests that TRAF2 and/or TRAF6 are required for recruitment of PI3K or Akt into TNFR signalosomes, but whether these TRAF molecules directly bind to PI3K or Akt is not clear. In overexpression studies, and in MEFs stimulated with IGF-1 or IL-1, TRAF6 was precipitated with Akt, and furthermore TRAF6 induced K63-linked ubiquitination and membrane localization of Akt (Yang et al., 2009). Thus it is possible that this is also a primary activity when TRAF6 is recruited to a TNFR molecule. However, not all TNFR family members that have been reported to promote PI3K and Akt activation appear to bind or recruit TRAF6, but they may all interact with TRAF2. This implies that TRAF2 could be the crucial adaptor in some cases, but whether TRAF2 possesses the same activity as TRAF6 in being able to complex with, and ubiquitinate, Akt is presently unknown.

In T cells, TNFR family molecules are crucial for clonal expansion and survival, and for the generation of T cell memory, and increasing evidence suggests these functional effects are in part mediated by enhancing antigen-initiated PI3K and Akt activity. Some TNFR family molecules in non-T cells appear to have the capacity to activate PI3K and Akt without signaling from other receptors, suggesting they may directly phosphorylate PI3K and/or recruit other kinases that can perform this function, and they may also recruit kinases such as PDK1 that phosphorylate Akt. In contrast, most evidence suggests that in T cells the ability of TNFR molecules to promote phosphorylation of PI3K and Akt is restricted unless the TCR recognizes antigen. This makes sense as T cells are governed by many checkpoints that limit their response in an attempt to control autoreactivity. Thus, it is likely that the higher-order TNFR-TRAF superclusters induced within lipid-rich microdomains of T cells then allow the efficient accumulation of PI3K and Akt in concentrated depots in close proximity to the TCR signalosome, and the function of this would be to either relieve a molecular checkpoint that limits Akt phosphorylation, or would be to simply provide more molecules of Akt available to the TCR. Further work in this area is required to fully understand the nature of TNFR signalosomes and how they may differ from molecule to molecule and in T cells versus other cell types.

## Conflict of Interest Statement

The authors declare that the research was conducted in the absence of any commercial or financial relationships that could be construed as a potential conflict of interest.
